# U.S. congressional district cancer death rates

**DOI:** 10.1186/1476-072X-5-28

**Published:** 2006-06-23

**Authors:** Yongping Hao, Elizabeth M Ward, Ahmedin Jemal, Linda W Pickle, Michael J Thun

**Affiliations:** 1 American Cancer Society, 1599 Clifton Road, NE, Atlanta, Georgia, USA; 2 NCI/DCCPS, 6116 Executive Blvd., Suite 504, Bethesda, Maryland, USA

## Abstract

**Background:**

Geographic patterns of cancer death rates in the U.S. have customarily been presented by county or aggregated into state economic or health service areas. Herein, we present the geographic patterns of cancer death rates in the U.S. by congressional district. Many congressional districts do not follow state or county boundaries. However, counties are the smallest geographical units for which death rates are available. Thus, a method based on the hierarchical relationship of census geographic units was developed to estimate age-adjusted death rates for congressional districts using data obtained at county level. These rates may be useful in communicating to legislators and policy makers about the cancer burden and potential impact of cancer control in their jurisdictions.

**Results:**

Mortality data were obtained from the National Center for Health Statistics (NCHS) for 1990–2001 for 50 states, the District of Columbia, and all counties. We computed annual average age-adjusted death rates for all cancer sites combined, the four major cancers (lung and bronchus, prostate, female breast, and colorectal cancer) and cervical cancer. Cancer death rates varied widely across congressional districts for all cancer sites combined, for the four major cancers, and for cervical cancer. When examined at the national level, broad patterns of mortality by sex, race and region were generally similar with those previously observed based on county and state economic area.

**Conclusion:**

We developed a method to generate cancer death rates by congressional district using county-level mortality data. Characterizing the cancer burden by congressional district may be useful in promoting cancer control and prevention programs, and persuading legislators to enact new cancer control programs and/or strengthening existing ones. The method can be applied to state legislative districts and other analyses that involve data aggregation from different geographic units.

## Background

Cancer death rates presented by geographic boundaries such as state and county, state economic areas, and health service areas have been useful in monitoring temporal trends in allocating public health resources [1,2], and in some instances, in generating etiological hypotheses. These rates are less useful for communicating to legislators and policy makers whose jurisdictions are not defined by state or county boundaries. There have been no published studies that attempted to measure cancer death rates within congressional districts.

Public policy and legislation play a critically important role in efforts to reduce the burden of cancer. For example, the American Cancer Society estimates that in 2006 about 170,000 of the 564,830 cancer deaths are expected to be caused by tobacco use alone [3]. Policy measures that are proven to reduce smoking prevalence include excise taxes and funding for state comprehensive tobacco control programs [4-6]. Declines in smoking prevalence among men as a result of public health efforts have had a major influence on the declines in cancer mortality in the last decade.

We present a method to calculate cancer death rates according to congressional district that may be useful in advocating for legislative initiatives and funding for cancer research and prevention programs.

## Results and discussion

**Figure 1:**
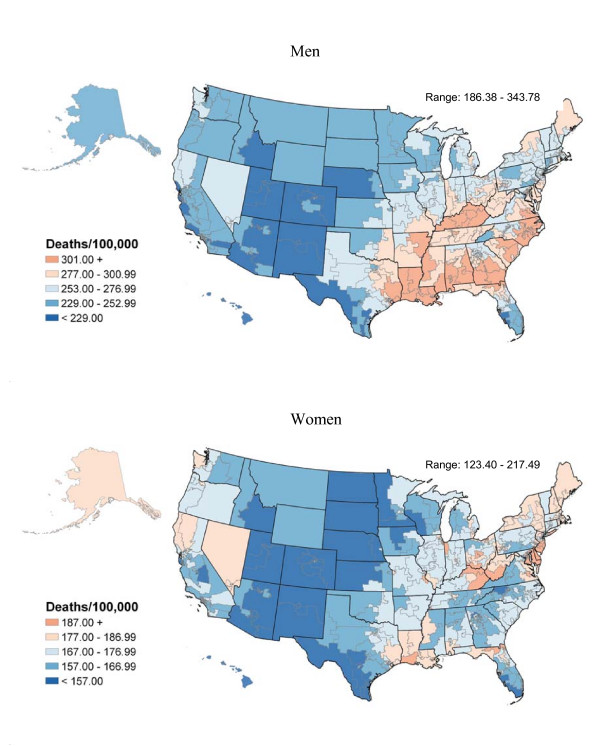
All cancers combined death rates per 100,000 person-years by congressional district (age-adjusted 2000 US population), 1990–2001.

**Figure 2:**
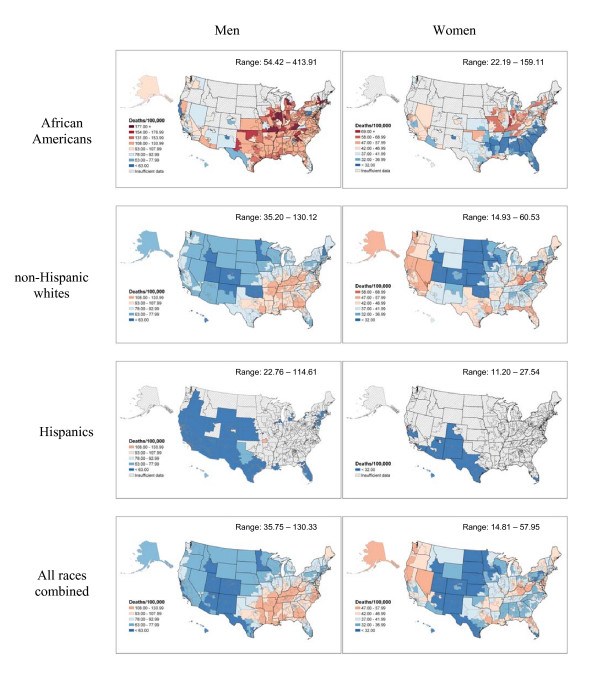
Lung cancer death rates per 100,000 person-years by congressional district (age-adjusted 2000 US population), 1990–2001.

**Figure 3:**
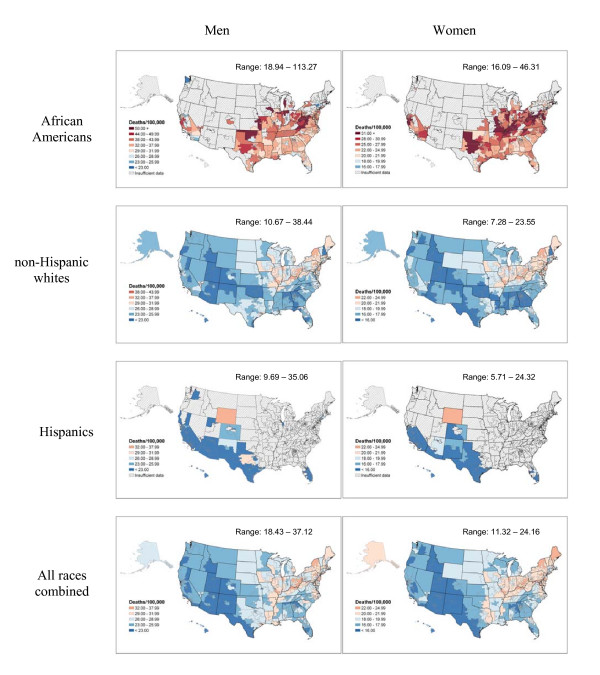
Colorectal cancer death rates per 100,000 person-years by congressional district (age-adjusted 2000 US population), 1990–2001.

**Figure 4:**
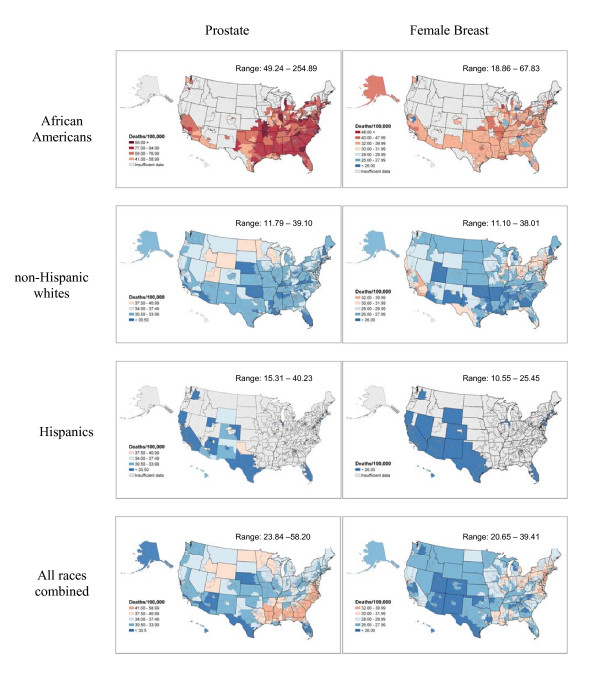
Prostate, female breast cancer death rates per 100,000 person-years by congressional district (age-adjusted 2000 US population), 1990–2001.

**Figure 5:**
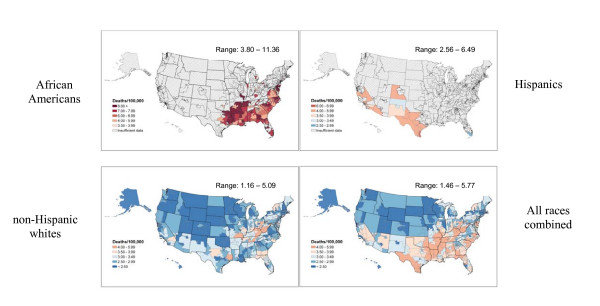
Cervical cancer death rates per 100,000 person-years by congressional district (age-adjusted 2000 US population), 1990–2001.

Maps of cancer death rates by congressional district were prepared for men and women, for all races combined, and for African Americans, non-Hispanic whites, and Hispanics (Figures 1, 2, 3, 4, 5); Hispanics are not mutually exclusive of whites and African Americans. Regional patterns of cancer mortality for African Americans and non-Hispanic whites were compared to previously published maps based on counties and state economic areas [1]. Although maps of cancer mortality by congressional district were also prepared for Hispanics, regional patterns are difficult to interpret because of insufficient data to calculate rates for most parts of the country. When examined at the national level, broad patterns of mortality for African Americans and non-Hispanic whites by sex and region were consistent with those previously observed [1]. Geographic variations in cancer death rates may reflect, in part, regional variations in risk factors such as smoking and obesity, early detection and screening, and access to and utilization of medical services.

Figure 1 shows geographic patterns of death rates for all cancer sites combined by congressional district in the United States. In men, rates range from 186.3 in Utah congressional district #3 to 343.7 in District of Columbia (Table 1) and in women, from 123.4 in Utah congressional district #1 to 217.4 in Pennsylvania congressional district #2 (Table 2). Generally, the patterns for all cancer sites combined are strikingly similar to those for lung cancer (Figure 2), reflecting the importance of lung cancer as a cause of cancer death, and the strong association of lung and cancers of several other sites with tobacco smoking. Lung cancer death rates in all races combined range from 35.7 in Utah congressional district #1 to 130.3 in Kentucky congressional district #5 for men and from 14.8 in Utah congressional district #3 to 57.9 in Kentucky congressional district #5 for women. Lung cancer death rates are the highest in congressional districts in Appalachia and the south among non-Hispanic white men and in the Midwest and the south among African American men. In contrast, among women, rates are the highest in congressional districts in the Midwest among African Americans and in the west, Appalachia, and the coastal south among non-Hispanic whites. Historically, smoking was more common in the south among men and in the west among women, especially among whites [7]. Although patterns of lung cancer mortality in the 1990's primarily reflect smoking patterns in the 1950's and 1960's, the burden of death from all cancers and lung cancer by congressional district can be used to illustrate the importance of tobacco control measures as well as to document local needs for cancer treatment and associated services.

**Table 1:** Age-adjusted death rates, all cancers combined, for US men by congressional district (CD), 1990–2001

**State**	**CD**	**Rate**	**State**	**CD**	**Rate**	**State**	**CD**	**Rate**	**State**	**CD**	**Rate**
AL	0101	311.55	FL	1223	233.18	MN	2705	246.79	OR	4102	245.13
AL	0102	309.74	FL	1224	262.08	MN	2706	243.38	OR	4103	270.72
AL	0103	312.74	FL	1225	231.74	MN	2707	235.05	OR	4104	246.92
AL	0104	290.71	GA	1301	306.92	MN	2708	250.08	OR	4105	246.09
AL	0105	262.11	GA	1302	318.36	MS	2801	299.09	PA	4201	341.70
AL	0106	286.12	GA	1303	310.67	MS	2802	330.08	PA	4202	343.25
AL	0107	307.46	GA	1304	256.56	MS	2803	299.83	PA	4203	262.65
AK	0299	248.48	GA	1305	283.68	MS	2804	314.84	PA	4204	279.79
AZ	0401	205.84	GA	1306	271.97	MO	2901	282.13	PA	4205	250.82
AZ	0402	239.41	GA	1307	253.45	MO	2902	256.11	PA	4206	251.69
AZ	0403	229.35	GA	1308	283.26	MO	2903	298.52	PA	4207	276.22
AZ	0404	229.35	GA	1309	276.76	MO	2904	264.86	PA	4208	272.61
AZ	0405	229.35	GA	1310	276.81	MO	2905	277.15	PA	4209	253.47
AZ	0406	227.76	GA	1311	290.20	MO	2906	263.57	PA	4210	260.76
AZ	0407	211.10	GA	1312	295.19	MO	2907	272.91	PA	4211	274.08
AZ	0408	234.26	GA	1313	267.16	MO	2908	290.16	PA	4212	268.01
AR	0501	307.86	HI	1501	202.59	MO	2909	264.05	PA	4213	295.64
AR	0502	292.46	HI	1502	202.59	MT	3099	248.52	PA	4214	288.08
AR	0503	264.97	ID	1601	234.87	NE	3101	242.74	PA	4215	253.36
AR	0504	296.35	ID	1602	221.35	NE	3102	267.93	PA	4216	244.42
CA	0601	257.81	IL	1701	287.98	NE	3103	226.06	PA	4217	266.93
CA	0602	266.90	IL	1702	287.63	NV	3201	268.19	PA	4218	277.63
CA	0603	245.75	IL	1703	287.98	NV	3202	254.67	PA	4219	252.99
CA	0604	236.01	IL	1704	287.98	NV	3203	268.19	RI	4401	276.83
CA	0605	245.61	IL	1705	287.98	NH	3301	270.77	RI	4402	278.12
CA	0606	227.02	IL	1706	256.03	NH	3302	266.04	SC	4501	293.71
CA	0607	244.64	IL	1707	287.98	NJ	3401	292.38	SC	4502	279.65
CA	0608	244.76	IL	1708	265.27	NJ	3402	290.30	SC	4503	283.26
CA	0609	246.04	IL	1709	287.98	NJ	3403	277.44	SC	4504	280.26
CA	0610	242.33	IL	1710	269.31	NJ	3404	275.30	SC	4505	311.21
CA	0611	242.00	IL	1711	272.33	NJ	3405	259.29	SC	4506	313.81
CA	0612	232.65	IL	1712	296.31	NJ	3406	273.02	SD	4699	246.34
CA	0613	246.04	IL	1713	257.26	NJ	3407	260.46	TN	4701	288.63
CA	0614	216.61	IL	1714	248.91	NJ	3408	279.73	TN	4702	281.01
CA	0615	208.66	IL	1715	267.45	NJ	3409	260.33	TN	4703	293.12
CA	0616	208.66	IL	1716	266.46	NJ	3410	285.53	TN	4704	299.25
CA	0617	220.87	IL	1717	273.62	NJ	3411	253.50	TN	4705	301.32
CA	0618	248.61	IL	1718	274.38	NJ	3412	271.16	TN	4706	282.64
CA	0619	239.15	IL	1719	275.28	NJ	3413	283.59	TN	4707	295.64
CA	0620	235.22	IN	1801	297.56	NM	3501	224.30	TN	4708	299.44
CA	0621	231.25	IN	1802	273.64	NM	3502	227.97	TN	4709	323.86
CA	0622	241.10	IN	1803	264.13	NM	3503	205.63	TX	4801	298.28
CA	0623	216.41	IN	1804	278.64	NY	3601	272.33	TX	4802	302.76
CA	0624	218.17	IN	1805	265.45	NY	3602	269.70	TX	4803	251.80
CA	0625	234.12	IN	1806	271.20	NY	3603	245.27	TX	4804	280.20
CA	0626	239.12	IN	1807	310.26	NY	3604	236.48	TX	4805	296.25
CA	0627	229.74	IN	1808	287.76	NY	3605	225.59	TX	4806	281.01
CA	0628	229.74	IN	1809	286.44	NY	3606	222.78	TX	4807	277.95
CA	0629	229.74	IA	1901	259.56	NY	3607	247.39	TX	4808	282.93
CA	0630	229.74	IA	1902	250.56	NY	3608	247.21	TX	4809	302.08
CA	0631	229.74	IA	1903	256.54	NY	3609	229.07	TX	4810	242.29
CA	0632	229.74	IA	1904	242.92	NY	3610	242.88	TX	4811	272.71
CA	0633	229.74	IA	1905	244.45	NY	3611	242.94	TX	4812	272.87
CA	0634	229.74	KS	2001	236.43	NY	3612	240.42	TX	4813	267.39
CA	0635	229.74	KS	2002	254.68	NY	3613	263.79	TX	4814	267.50
CA	0636	229.74	KS	2003	243.40	NY	3614	241.66	TX	4815	200.38
CA	0637	229.74	KS	2004	259.82	NY	3615	251.70	TX	4816	223.16
CA	0638	229.74	KY	2101	301.17	NY	3616	267.24	TX	4817	270.88
CA	0639	229.74	KY	2102	302.60	NY	3617	255.21	TX	4818	277.95
CA	0640	224.83	KY	2103	319.57	NY	3618	245.32	TX	4819	258.34
CA	0641	248.53	KY	2104	311.74	NY	3619	263.83	TX	4820	252.64
CA	0642	232.32	KY	2105	314.33	NY	3620	266.28	TX	4821	247.33
CA	0643	253.34	KY	2106	306.21	NY	3621	267.14	TX	4822	263.97
CA	0644	225.41	LA	2201	313.23	NY	3622	270.59	TX	4823	226.97
CA	0645	225.51	LA	2202	341.56	NY	3623	278.23	TX	4824	275.61
CA	0646	226.08	LA	2203	317.11	NY	3624	257.38	TX	4825	276.05
CA	0647	224.82	LA	2204	314.28	NY	3625	266.60	TX	4826	250.08
CA	0648	224.82	LA	2205	321.98	NY	3626	270.45	TX	4827	229.00
CA	0649	232.00	LA	2206	302.08	NY	3627	271.37	TX	4828	231.66
CA	0650	235.70	LA	2207	307.17	NY	3628	268.37	TX	4829	277.95
CA	0651	235.62	ME	2301	272.57	NY	3629	268.26	TX	4830	279.05
CA	0652	235.70	ME	2302	291.59	NC	3701	325.75	TX	4831	258.48
CA	0653	235.70	MD	2401	293.67	NC	3702	307.11	TX	4832	279.05
CO	0801	247.17	MD	2402	300.57	NC	3703	312.42	UT	4901	188.85
CO	0802	216.40	MD	2403	306.03	NC	3704	276.61	UT	4902	194.50
CO	0803	218.01	MD	2404	261.33	NC	3705	270.43	UT	4903	186.38
CO	0804	217.45	MD	2405	293.74	NC	3706	269.53	VT	5099	262.46
CO	0805	230.10	MD	2406	268.50	NC	3707	303.46	VA	5101	294.08
CO	0806	205.15	MD	2407	331.59	NC	3708	295.65	VA	5102	291.22
CO	0807	223.10	MD	2408	212.85	NC	3709	280.84	VA	5103	335.68
CT	0901	252.15	MA	2501	266.20	NC	3710	283.71	VA	5104	321.70
CT	0902	255.68	MA	2502	273.91	NC	3711	251.18	VA	5105	278.86
CT	0903	253.05	MA	2503	272.89	NC	3712	273.38	VA	5106	270.54
CT	0904	237.15	MA	2504	275.28	NC	3713	274.86	VA	5107	289.48
CT	0905	246.80	MA	2505	268.96	ND	3899	243.02	VA	5108	228.11
DE	1099	289.44	MA	2506	270.11	OH	3901	295.76	VA	5109	274.86
DC	1198	343.78	MA	2507	271.96	OH	3902	293.68	VA	5110	258.25
FL	1201	287.59	MA	2508	295.36	OH	3903	284.95	VA	5111	231.79
FL	1202	287.22	MA	2509	283.39	OH	3904	274.64	WA	5301	245.00
FL	1203	285.46	MA	2510	269.84	OH	3905	262.93	WA	5302	234.80
FL	1204	316.89	MI	2601	261.34	OH	3906	287.57	WA	5303	255.32
FL	1205	256.17	MI	2602	248.17	OH	3907	276.90	WA	5304	240.69
FL	1206	281.19	MI	2603	245.36	OH	3908	271.26	WA	5305	246.75
FL	1207	262.33	MI	2604	260.27	OH	3909	287.34	WA	5306	260.08
FL	1208	262.72	MI	2605	278.91	OH	3910	293.92	WA	5307	239.57
FL	1209	265.45	MI	2606	266.81	OH	3911	293.92	WA	5308	244.02
FL	1210	249.68	MI	2607	263.88	OH	3912	281.32	WA	5309	249.13
FL	1211	277.62	MI	2608	253.12	OH	3913	277.94	WV	5401	278.03
FL	1212	265.00	MI	2609	247.44	OH	3914	266.04	WV	5402	296.32
FL	1213	225.69	MI	2610	272.66	OH	3915	293.41	WV	5403	298.58
FL	1214	215.92	MI	2611	284.77	OH	3916	259.50	WI	5501	265.84
FL	1215	252.94	MI	2612	263.76	OH	3917	272.68	WI	5502	235.97
FL	1216	236.00	MI	2613	300.81	OH	3918	280.22	WI	5503	244.95
FL	1217	238.61	MI	2614	300.81	OK	4001	270.44	WI	5504	285.86
FL	1218	239.87	MI	2615	272.06	OK	4002	295.23	WI	5505	247.27
FL	1219	225.47	MN	2701	234.69	OK	4003	252.79	WI	5506	248.00
FL	1220	241.08	MN	2702	232.60	OK	4004	263.30	WI	5507	253.68
FL	1221	237.96	MN	2703	246.78	OK	4005	273.49	WI	5508	252.81
FL	1222	228.26	MN	2704	253.04	OR	4101	239.29	WY	5699	240.61

**Table 2:** Age-adjusted death rates, all cancers combined, for US women by congressional district (CD), 1990–2001

**State**	**CD**	**Rate**	**State**	**CD**	**Rate**	**State**	**CD**	**Rate**	**State**	**CD**	**Rate**
AL	0101	178.15	FL	1223	166.84	MN	2705	167.95	OR	4102	167.81
AL	0102	169.16	FL	1224	171.40	MN	2706	159.96	OR	4103	181.38
AL	0103	173.01	FL	1225	148.24	MN	2707	149.49	OR	4104	175.60
AL	0104	160.11	GA	1301	171.36	MN	2708	167.49	OR	4105	170.49
AL	0105	158.39	GA	1302	164.99	MS	2801	163.41	PA	4201	216.57
AL	0106	166.72	GA	1303	160.00	MS	2802	178.71	PA	4202	217.49
AL	0107	173.12	GA	1304	158.33	MS	2803	162.39	PA	4203	171.06
AK	0299	177.59	GA	1305	174.21	MS	2804	173.19	PA	4204	177.43
AZ	0401	150.54	GA	1306	168.46	MO	2901	184.42	PA	4205	167.87
AZ	0402	160.42	GA	1307	156.76	MO	2902	172.86	PA	4206	170.48
AZ	0403	155.51	GA	1308	166.01	MO	2903	191.43	PA	4207	185.30
AZ	0404	155.51	GA	1309	160.49	MO	2904	167.09	PA	4208	182.33
AZ	0405	155.51	GA	1310	158.41	MO	2905	180.75	PA	4209	162.09
AZ	0406	154.84	GA	1311	168.71	MO	2906	167.65	PA	4210	169.23
AZ	0407	143.81	GA	1312	169.92	MO	2907	166.90	PA	4211	175.65
AZ	0408	155.45	GA	1313	166.59	MO	2908	173.10	PA	4212	169.55
AR	0501	176.39	HI	1501	132.18	MO	2909	168.23	PA	4213	195.46
AR	0502	167.22	HI	1502	132.18	MT	3099	164.72	PA	4214	185.54
AR	0503	159.68	ID	1601	159.32	NE	3101	154.37	PA	4215	167.46
AR	0504	171.75	ID	1602	145.79	NE	3102	172.54	PA	4216	166.84
CA	0601	180.93	IL	1701	187.65	NE	3103	148.99	PA	4217	171.04
CA	0602	179.84	IL	1702	187.39	NV	3201	185.55	PA	4218	179.77
CA	0603	173.30	IL	1703	187.65	NV	3202	178.47	PA	4219	164.61
CA	0604	171.91	IL	1704	187.65	NV	3203	185.55	RI	4401	176.99
CA	0605	174.43	IL	1705	187.65	NH	3301	184.05	RI	4402	181.40
CA	0606	174.68	IL	1706	171.34	NH	3302	177.78	SC	4501	168.40
CA	0607	172.79	IL	1707	187.65	NJ	3401	197.38	SC	4502	169.68
CA	0608	160.82	IL	1708	183.94	NJ	3402	194.20	SC	4503	160.68
CA	0609	171.62	IL	1709	187.65	NJ	3403	187.01	SC	4504	163.56
CA	0610	171.36	IL	1710	184.25	NJ	3404	189.04	SC	4505	170.43
CA	0611	166.00	IL	1711	176.79	NJ	3405	181.48	SC	4506	170.50
CA	0612	163.35	IL	1712	182.42	NJ	3406	189.45	SD	4699	155.91
CA	0613	171.63	IL	1713	170.69	NJ	3407	175.40	TN	4701	163.70
CA	0614	155.60	IL	1714	173.48	NJ	3408	186.04	TN	4702	166.03
CA	0615	150.41	IL	1715	169.81	NJ	3409	179.94	TN	4703	170.24
CA	0616	150.41	IL	1716	173.31	NJ	3410	190.31	TN	4704	166.86
CA	0617	159.08	IL	1717	169.35	NJ	3411	178.32	TN	4705	181.74
CA	0618	167.37	IL	1718	175.46	NJ	3412	185.32	TN	4706	166.05
CA	0619	160.90	IL	1719	171.91	NJ	3413	185.44	TN	4707	171.72
CA	0620	160.07	IN	1801	187.73	NM	3501	152.60	TN	4708	172.99
CA	0621	155.17	IN	1802	174.13	NM	3502	148.21	TN	4709	191.57
CA	0622	167.90	IN	1803	171.04	NM	3503	145.39	TX	4801	170.48
CA	0623	156.79	IN	1804	175.19	NY	3601	193.45	TX	4802	179.62
CA	0624	159.18	IN	1805	174.37	NY	3602	192.13	TX	4803	158.43
CA	0625	165.29	IN	1806	173.27	NY	3603	180.21	TX	4804	171.21
CA	0626	167.46	IN	1807	195.50	NY	3604	175.92	TX	4805	174.87
CA	0627	163.44	IN	1808	174.00	NY	3605	159.07	TX	4806	173.74
CA	0628	163.44	IN	1809	174.32	NY	3606	154.59	TX	4807	174.78
CA	0629	163.44	IA	1901	167.19	NY	3607	167.00	TX	4808	175.14
CA	0630	163.44	IA	1902	160.01	NY	3608	169.61	TX	4809	184.12
CA	0631	163.44	IA	1903	166.60	NY	3609	157.90	TX	4810	161.79
CA	0632	163.44	IA	1904	155.81	NY	3610	165.33	TX	4811	162.37
CA	0633	163.44	IA	1905	158.63	NY	3611	165.35	TX	4812	173.24
CA	0634	163.44	KS	2001	150.79	NY	3612	164.75	TX	4813	166.63
CA	0635	163.44	KS	2002	164.11	NY	3613	180.01	TX	4814	161.32
CA	0636	163.44	KS	2003	162.29	NY	3614	168.07	TX	4815	130.06
CA	0637	163.44	KS	2004	167.47	NY	3615	173.80	TX	4816	150.47
CA	0638	163.44	KY	2101	169.41	NY	3616	175.64	TX	4817	163.78
CA	0639	163.44	KY	2102	175.24	NY	3617	173.76	TX	4818	174.78
CA	0640	158.89	KY	2103	193.34	NY	3618	170.09	TX	4819	158.33
CA	0641	171.99	KY	2104	188.93	NY	3619	184.37	TX	4820	159.07
CA	0642	162.99	KY	2105	194.13	NY	3620	182.40	TX	4821	156.84
CA	0643	173.93	KY	2106	182.99	NY	3621	181.17	TX	4822	163.94
CA	0644	162.59	LA	2201	185.99	NY	3622	184.58	TX	4823	145.28
CA	0645	163.17	LA	2202	195.03	NY	3623	181.55	TX	4824	173.40
CA	0646	160.07	LA	2203	183.13	NY	3624	172.75	TX	4825	173.48
CA	0647	158.89	LA	2204	181.02	NY	3625	177.64	TX	4826	166.78
CA	0648	158.89	LA	2205	178.98	NY	3626	178.41	TX	4827	145.93
CA	0649	165.98	LA	2206	180.49	NY	3627	181.68	TX	4828	145.40
CA	0650	167.74	LA	2207	187.87	NY	3628	178.66	TX	4829	174.78
CA	0651	164.39	ME	2301	184.93	NY	3629	181.02	TX	4830	173.69
CA	0652	167.74	ME	2302	183.09	NC	3701	174.50	TX	4831	160.07
CA	0653	167.74	MD	2401	188.54	NC	3702	165.66	TX	4832	173.69
CO	0801	162.28	MD	2402	192.89	NC	3703	174.10	UT	4901	123.40
CO	0802	153.27	MD	2403	196.95	NC	3704	170.87	UT	4902	131.73
CO	0803	147.33	MD	2404	174.28	NC	3705	155.94	UT	4903	127.35
CO	0804	147.02	MD	2405	189.44	NC	3706	162.13	VT	5099	172.62
CO	0805	153.67	MD	2406	169.34	NC	3707	168.11	VA	5101	180.85
CO	0806	153.08	MD	2407	205.58	NC	3708	169.12	VA	5102	184.69
CO	0807	153.73	MD	2408	150.96	NC	3709	168.36	VA	5103	197.48
CT	0901	167.11	MA	2501	174.47	NC	3710	157.81	VA	5104	186.12
CT	0902	169.98	MA	2502	178.16	NC	3711	158.78	VA	5105	163.70
CT	0903	172.06	MA	2503	178.00	NC	3712	166.97	VA	5106	163.67
CT	0904	167.64	MA	2504	179.23	NC	3713	165.79	VA	5107	176.21
CT	0905	167.56	MA	2505	179.67	ND	3899	156.30	VA	5108	165.17
DE	1099	190.49	MA	2506	179.61	OH	3901	193.84	VA	5109	166.02
DC	1198	203.38	MA	2507	181.29	OH	3902	190.45	VA	5110	170.94
FL	1201	170.38	MA	2508	190.60	OH	3903	185.18	VA	5111	168.97
FL	1202	177.20	MA	2509	188.59	OH	3904	171.76	WA	5301	171.78
FL	1203	180.69	MA	2510	184.62	OH	3905	164.79	WA	5302	169.26
FL	1204	187.94	MI	2601	170.39	OH	3906	179.70	WA	5303	176.09
FL	1205	165.25	MI	2602	161.41	OH	3907	182.23	WA	5304	163.04
FL	1206	174.62	MI	2603	163.09	OH	3908	177.92	WA	5305	166.08
FL	1207	170.67	MI	2604	164.71	OH	3909	184.70	WA	5306	180.48
FL	1208	172.08	MI	2605	177.98	OH	3910	188.77	WA	5307	166.68
FL	1209	168.29	MI	2606	172.69	OH	3911	188.77	WA	5308	169.06
FL	1210	159.50	MI	2607	173.30	OH	3912	187.23	WA	5309	171.64
FL	1211	172.59	MI	2608	169.43	OH	3913	180.65	WV	5401	178.91
FL	1212	160.52	MI	2609	171.89	OH	3914	177.31	WV	5402	186.23
FL	1213	150.69	MI	2610	175.35	OH	3915	191.61	WV	5403	191.78
FL	1214	144.66	MI	2611	185.66	OH	3916	168.52	WI	5501	173.85
FL	1215	166.13	MI	2612	173.14	OH	3917	174.30	WI	5502	160.12
FL	1216	159.77	MI	2613	191.34	OH	3918	176.73	WI	5503	156.93
FL	1217	155.30	MI	2614	191.34	OK	4001	174.42	WI	5504	183.35
FL	1218	152.52	MI	2615	181.41	OK	4002	175.25	WI	5505	164.99
FL	1219	163.40	MN	2701	150.21	OK	4003	157.63	WI	5506	163.77
FL	1220	167.15	MN	2702	161.35	OK	4004	162.63	WI	5507	158.81
FL	1221	152.17	MN	2703	167.91	OK	4005	175.18	WI	5508	157.81
FL	1222	164.56	MN	2704	172.77	OR	4101	169.53	WY	5699	164.81

Historically, female breast cancer death rates have been elevated in the Northeastern and North Central regions; North-South differences have diminished over time as female breast cancer death rates decreased in the Northeast but increased in the South [8]. For all races combined, female breast cancer death rates vary from 20.6 in Hawaii to 39.4 in District of Columbia. Among African American women, breast cancer death rates are highest in congressional districts in the south, Midwest, and west coast, while among non-Hispanic whites, breast cancer mortality is highest in congressional districts in the Northeast and west coast (Figure 4, right panel). Patterns of breast cancer mortality partly reflect the influence of known risk factors as well as access to and utilization of cancer screening and treatment. Important cancer control measures include access to mammography for the uninsured and under-insured, and availability of Medicaid coverage for diagnosis and treatment.

Colorectal cancer death rates are highest overall in the Northeast and parts of the South and Midwest. Generally, death rates range from 18.4 in Texas congressional district #15 to 37.1 in Pennsylvania congressional district #1 for men and from 11.3 in Texas congressional district #15 to 24.1 in District of Columbia for women (Figure 3). Although a strong geographic pattern for colorectal cancer mortality has existed since the 1950's, the reasons are not well-understood [1]. The current priority for colorectal cancer control is to increase the proportion of individuals over 50 who receive recommended screening tests. Illustrating colorectal cancer mortality by legislative district may be influential in encouraging legislative support for mandated insurance coverage of colorectal screening tests and for programs to provide testing for the uninsured and under-insured.

For all races combined, prostate cancer death rates range from 23.8 in Texas congressional district #15 and Hawaii to 58.2 in District of Columbia. Generally, rates are highest in congressional districts in the mid-Atlantic and Southern coastal areas, reflecting in large part the higher proportion of the African American men in the population of these areas (Figure 4, left panel). Death rates for African American men are more than twice the rates for non-Hispanic white men, reflecting higher incidence, later stage at diagnosis and poorer survival among African American men. Among non-Hispanic whites, rates are highest in congressional districts in the Rocky Mountain region; high rate (40.2) is observed in Hispanics in Texas congressional district #13. A recent study suggested that 10% to 30% of the geographic variation in prostate cancer death rates might relate to variations in access to medical care [9]. Although cancer control measures for prostate cancer are less well-defined than measures for some other cancer sites, illustrating prostate cancer mortality by congressional district may be helpful in advocating for funding of research on the prevention, early detection and treatment of prostate cancer and highlighting the importance of access to medical care for African American men.

Mortality from cervical cancer in all races combined is highest in congressional districts in Appalachia, in the South and parts of the Southwest, with rates ranging from 1.4 in Minnesota congressional district #2 to 5.7 in New York congressional district #16 (Figure 5). Among African American women, rates are highest in congressional districts in the south and southeast, among non-Hispanic whites, rates are highest in congressional districts in Appalachia, and in Hispanics rates are highest in congressional districts in the coastal parts of California and Texas and in Colorado congressional district #3. Important cancer control measures include access to Pap tests for the uninsured and under-insured, and availability of Medicaid coverage for diagnosis and treatment.

## Conclusion

The cancer mortality patterns by congressional district are generally similar to the patterns seen using other geographic boundaries. However, the patterns by congressional district may be useful to cancer control advocates to illustrate the importance of cancer control measures (prevention, early detection, and treatment) for their constituents. The method can be applied to state legislative districts and other analyses that involve data aggregation from different geographic units. Further research is needed to validate the estimates using mortality data geocoded to the lower geographic level such as block.

## Methods

### Death rates for U.S. states and counties

Mortality data were obtained from the National Center for Health Statistics (NCHS). We computed annual average age-adjusted death rates for all cancer sites combined, the four major cancers (lung and bronchus, prostate, female breast, and colorectal cancer) and cervical cancer from 1990–2001 for 50 states, District of Columbia, and all counties using SEER*Stat [10]. Death rates, counts (number of deaths), and populations for counties were directly obtained for men and women, for all races combined, and for African Americans, non-Hispanic whites, and Hispanics. Except for the years of 1990 and 2000, the intercensal populations computed by the Census Bureau were used to obtain the total populations for the study time period. Since county designation for Alaska and Hawaii was not available from NCHS, death rates for Alaska and Hawaii reflect state rates. Rates were standardized to the 2000 U.S. population and expressed per 100,000 person-years.

### Death rates for U.S. congressional districts

There are 436 (excluding Puerto Rico) federal congressional districts in the U.S. [11]. Among these, eight congressional districts followed state boundaries or their equivalent (Alaska, District of Columbia, Delaware, Montana, North Dakota, South Dakota, Vermont, and Wyoming). Further, since county-specific mortality data were not provided for Hawaii in SEER*Stat, we assigned the state death rate to both congressional districts. For congressional districts whose boundaries did not follow state and county boundaries (n = 426), death rates were calculated by assigning county-level age-adjusted death rates to census block and then aggregating death rates over blocks by congressional district using GIS [12] and SAS [13]. By doing so, we assume that blocks within a county have same death rates.

There are three major areal interpolation methods (area weighting, surface smoothing, and dasymetric technique) for generating estimates for target zones from data available for source zones when the two geographic units are not comparable. Areal weighting assumes that data are homogeneously distributed across geographic units, which is generally unrealistic; it also involves the direct superimposition of source zones and target zones [14], which often leads to a lot of geographic boundary-line discrepancies [15]. Surface smoothing models data available for source zones as a continuous surface across the adjacent zones, assuming that the density declines with distance, taking into account the proximity of neighboring centroids [16,17]. Dasymetric technique uses ancillary information to refine uneven data distributions across geographic units. Land cover from remote sensing [18] and the street layer [15,19] have been used as subzone ancillary information. A recent study uses parish level (the lowest administrative unit) population data to derive weights [20]. However, there is no universal rule to construct areal interpolation, and the best solution depends on various factors: the variables of interest, the spatial relationships between source zones and target zones, and the availability of ancillary information related to both.

In this study, we constructed a dasymetric method based on the hierarchical spatial relationships between blocks and counties and between blocks and congressional districts. Generally, congressional district and county share census block as a common basic spatial unit (Table 3) [21,22]. We used block level sex- and race- specific population to devise a dasymetric approach that assigns county-level measures such as cancer death rates to census block and then aggregates census blocks at the congressional district level, using block population as a weighting factor. We did not use area weighting because of its unrealistic homogeneity assumption and boundary-line discrepancies associated with direct superimposition of two incomparable geographic units. Surface smoothing gives reliable estimates when smoothness is the real property of the density. However, the occurrence of cancer rarely follows a smooth distance-decay surface because major risk factors that affect cancer occurrence do not have smooth paths from the centroid to its adjacent neighboring centroids.

**Table 3:** The hierarchical spatial relationships between blocks and counties and between blocks and congressional districts

**County**	**Block**	**Congressional district**
County A	Block A1	
	Block A2	
	Block A3	
		Congressional district #1
	...	
**County B**	**Block B1**	
	**Block B2**	
	**Block B3**	
	**...**	
County C	Block C1	Congressional district #2
	Block C2	
	Block C3	
	*...*	
**...**	**...**	...

To make the calculations, the following steps were taken:

1. The number of people living within each census block by sex and race was determined from the 2000 U.S. census (covering 42 states, 426 congressional districts). Therefore, block population is sex- and race- specific.

2. Block population was spatially assigned to congressional districts by block centroids.

3. The age-adjusted cancer death rates for counties by sex and race were assigned to block by county FIPS (Federal Information Processing Standards) codes; FIPS codes are a standardized set of numeric or alphabetic codes issued by the National Institute of Standards and Technology (NIST) to ensure uniform identification of geographic entities through all federal government agencies [23].

4. Cancer death rate for each congressional district by sex and race was calculated by aggregating sex- and race- specific cancer death rates over blocks. Taking non-Hispanic white men as an example, suppose that *r*_*i *_was the age-adjusted cancer death rate for block i (obtained from the corresponding county rate calculated from SEER*Stat). Suppose that *a*_*ij *_was the population of block i within district j, and that the population for district j, , were known. Then the aggregated cancer death rate for district j, *p*_*j*_, was the summation of *r*_*i*_, weighted by the proportion of block population within the district,. Other sex- and race-specific cancer death rates were calculated similarly.

5. The number of cancer deaths for each congressional district by sex and race was calculated by aggregating the sex- and race- specific number of cancer deaths over blocks. The number of cancer deaths for a block was the product of crude death rate for the block (inherited from the corresponding county, which is the number of deaths for the county divided by the county population) and the block population. Again, taking non-Hispanic white men as an example, suppose that *n*_*i *_and *c*_*i *_were the number of deaths and the population for the county to which block i belongs, the crude death rate for block i was . Given *a*_*ij *_was the population of block i within district j, then the number of deaths for block i within district j was *a*_*ij*_, and the aggregated number of deaths for district j was . Other sex- and race- specific number of cancer deaths were calculated in a similar way.

6. The aggregated cancer death rates and the number of cancer deaths for the congressional districts (n = 426) from step 4 & 5 were exported back to GIS and linked with the other ten congressional districts (Alaska, District of Columbia, Delaware, Montana, North Dakota, South Dakota, Vermont, Wyoming, and two Hawaii districts) for producing maps. The estimates of the number of deaths were not presented separately. Instead, they were used as the criteria when mapping death rates across congressional districts. Death rates based on the small number of deaths (< 20) for the study time period were considered not reliable and thus excluded.

7. Maps were generated using ArcGIS [12]. For all cancer sites combined and for each cancer site, the maps for all races combined were created by categorizing the rates into five groups. Cut points for the lowest and highest groups are approximately the 10th and 90th percentiles, except for cervical cancer which are 20th and 80th percentiles. Intervening groups are set at equal length between the lower bound cut point of 90th or 80th and the upper bound of 10th or 20th. Thus each interval represents the same absolute change over the middle range of rates, while the most extreme rates fall into the first and fifth categories. For each cancer site, to allow comparison among ethnic subgroups, the cut points for all races combined are used for race specific maps if rates are in the same range as those for all races combined. When the race specific rates fall out of the range of rates for all races combined, cut points for the exceeded portion are equally set at the length of rates in the highest category for all races combined. Cancer death rates based on the small number of deaths (< 20) are considered unstable and congressional districts with such rates are marked with hatches.

In describing the cancer burden by congressional district, we used direct age adjustment instead of indirect age adjustment because direct method is more statistically correct when the rates are being compared [24]. Direct age-adjusted death rates describe the cancer death rate each congressional district would have if it had the age-sex-race distribution of the U.S. in the year 2000. In so far as congressional districts have age-sex-race compositions different from the U.S. in 2000, the need for resources to eliminate disparities between districts might be more or less than that suggested by the results described in this paper.

## Competing interests

The author(s) declare that they have no competing interests.

## Authors' contributions

YH, EMW, and AJ conceived the analysis and wrote the final version of the manuscript. LWP provided technical support on the method and critically revised the manuscript. MJT conceptualized and critically revised the manuscript.

## Disclaimer

The views and opinions expressed in this article do not necessarily reflect those of the National Cancer Institute.
